# Honeybee Pollen From Southern Chile: Phenolic Profile, Antioxidant Capacity, Bioaccessibility, and Inhibition of DNA Damage

**DOI:** 10.3389/fphar.2022.775219

**Published:** 2022-03-07

**Authors:** Raquel Bridi, Javier Echeverría, Arturo Larena, Paula Nuñez Pizarro, Elias Atala, Adriano Costa De Camargo, Won Young Oh, Fereidoon Shahidi, Olga Garcia, Kong S. Ah-Hen, Gloria Montenegro

**Affiliations:** ^1^ Departamento de Química Farmacológica y Toxicológica, Facultad de Ciencias Químicas y Farmacéuticas, Universidad de Chile, Santiago, Chile; ^2^ Departamento de Ciencias del Ambiente, Facultad de Química y Biología, Universidad Santiago de Chile, Santiago, Chile; ^3^ Departamento de Farmacia, Facultad de Química y de Farmacia, Pontificia Universidad Católica de Chile, Santiago, Chile; ^4^ Doctorado en Ciencias de la Agricultura, Facultad de Agronomía e Ingeniería Forestal, Pontificia Universidad Católica de Chile, Santiago, Chile; ^5^ Centro Integrativo de Biología y Química Aplicada, Universidad Bernardo O’Higgins, Santiago, Chile; ^6^ Laboratory of Antioxidants, Nutrition and Food Technology Institute, University of Chile, Santiago, Chile; ^7^ Department of Biochemistry, Memorial University of Newfoundland, St. John’s, NL, Canada; ^8^ Instituto de Ciencia y Tecnología de los Alimentos, Facultad de Ciencias Agrarias, Universidad Austral de Chile, Valdivia, Chile

**Keywords:** honeybee pollen, phenolic compounds, antioxidant, bioaccessibility, DNA damage

## Abstract

Honeybee pollen (HBP) chemical composition is highly variable conforming to the floral and geographical origin of the pollen grains. The beneficial effects and functional properties of the HBP are well-known and have been mainly attributed to their high content of antioxidant polyphenols. In this work, twelve HBPs samples from the Southern region of Chile (X Región de Los Lagos) were characterized for the first time according to their botanical origin, phenolic composition, and antioxidant activity. The *in vitro* gastrointestinal digestion assay was done to simulate the human upper digestive tract. Selected honeybee pollen extracts (HBPEs) were assessed as bioaccessible fractions during an *in vitro* gastrointestinal digestion. Contents of phenolic compounds, antioxidant capacity, and recovery index of quercetin, myricetin, and cinnamic acid were monitored in different steps of gastrointestinal digestion. Furthermore, the protective effect of *in vitro* digested HBP towards DNA damage induced by peroxyl radicals was evaluated. The introduced species *Brassica rapa* L. (Brassicaceae), *Lotus pedunculatus* Cav. (Fabaceae), and *Ulex europaeus* L. (Fabaceae) predominated in all the HBPs analyzed, while the native species *Buddleja globosa* Hope (Scrophulariaceae), *Luma apiculata* (DC.) Burret (Myrtaceae), *Embothrium coccineum* J.R. Forst. & G. Forst. (Proteaceae) and *Eucryphia cordifolia* Cav. (Cunoniaceae) appeared less frequently. The content of polyphenols and antioxidant capacity in HBPEs achieved full bioaccessibility at the end of the intestinal digestion step. However, results obtained by a state-of-the-art technique (i.e. HPLC-DAD) demonstrated relatively low values of bioaccessible quercetin and cinnamic acid after the digestion process. In contrast, myricetin showed a high bioaccessibility in the intestinal digestion steps. The protective effect of *in vitro* digested HBP towards DNA damage induced by peroxyl radicals showed promising results (up to 91.2% protection). In conclusion, HBPs from the X Region de Los Lagos are rich sources of phenolic antioxidants that protect DNA from strand breakage. Therefore, the potential of HBPEs in preventing gastric and/or intestinal cancer should be further considered.

## 1 Introduction

Honeybee pollen (HBP) loads are a mix of flower pollen from different plant species adhered to by nectar and enzymes secreted by salivary glands of honeybees. The composition of HBP is quite variable and depends on ecological habitat, geographic origin, or even season (Denisow and Denisow-Pietrzyk, 2016; Ares et al., 2018; Bridi et al., 2019). It contains polysaccharides, lipids, proteins, aminoacids, and simple sugars. Moreover, it is a source of minerals (Cu, Fe, Zn, K, Na), vitamins, (β-carotene, tocopherol, niacin, thiamine, biotin, folic acid), and a variety of secondary metabolites such as terpenes, carotenoids, and phenolic compounds (Campos et al., 2008; Komosinska-Vassev et al., 2015).

Phenolic compounds, including flavonoids and phenolic acids, are recognized as important natural antioxidants, also playing a key role in a wide variety of biological and/or pharmacological properties such as anti-inflammatory, anticancer, antibacterial, antiallergic, antiviral, antithrombotic, hepatoprotective, and signalling molecules, among others (Kumar and Goel, 2019; Hızır-Kadı et al., 2020). The ingestion of phenolic compounds has been related to the reduced development of chronic diseases, as sustained by epidemiological studies (Ferrari and Torres, 2003; Torres and Farah, 2017; Soares et al., 2021).

Owing to their richness in nutrients, micronutrients, and abundance of bioactive compounds, honeybee products can be consumed as such (in the raw form). However, they are also accepted as “functional ingredients” since, beyond increasing the nutritional value of food products, they possess health-promoting properties (Cornara et al., 2017; Yücel et al., 2017; Kostić et al., 2021). Recently, hepatoprotective and anti-steatosis potential by reduction of lipid accumulation in a cellular model has been reported for Chilean honeybee pollen extracts (HBPEs). These results exhibited a positive correlation with the pollen’s quercetin concentration (Oyarzún et al., 2021). Additionally, HBP from the central zone of Chile showed a strong presence of phenolic compounds such as syringic and coumaric acids, and the flavonoids myricetin and quercetin, the latter, being proposed as a quality marker to indicate the quality of HBP from this region (Bridi et al., 2019).

Despite the great content of nutrients and active compounds that are found in HBP, the pollen cell walls consist of a series of stratified concentric layers that provide chemical resistance of the pollen and act as a shield that preserves these compounds (Fuenmayor et al., 2014; Zuluaga et al., 2014). This suggests that HBP for human consumption must undergo transformation processes to improve its digestibility and bioavailability (Zuluaga et al., 2014). Often, the total quantity of the bioactive compounds in functional plant foods does not reflect the amount absorbed by the human body. An *in vitro* digestion model has been designed to imitate the digestive processes in the human gastrointestinal tract in a simplified manner, hence providing significant information on the stability of phytochemicals of interest under simulated gastrointestinal conditions (Ah-Hen et al., 2018; Hızır-Kadı et al., 2020; Aylanc et al., 2021). This kind of information is crucial to anticipate the role of phytochemicals at local (gastrointestinal) and potential systemic levels (Adebooye et al., 2018; Fereidoon et al., 2019; Soares et al., 2021).

*In vivo* and *in vitro* studies support the role of flavonoids and phenolic acids in preventing DNA damage induced by reactive oxygen species (ROS) (Yonekura et al., 2016; Tasahil et al., 2019). DNA-damage signalling and repair are viewed as crucial pathways in cancer development and/or treatment (Amarowicz, 2016; de Camargo et al., 2018). Phenolics have significant free radical scavenging properties and may protect against cellular damage caused by free radicals, thereby providing precautions against various diseases. Phenolic compounds can inhibit free radical-induced DNA damage and suppress inflammation (Li et al., 2018). Humans do not have the ability to synthesize important antioxidants such as tocopherols, tocotrienols, and polyphenols, thus dietary antioxidants play an important role in maintaining human health (Nishikimi and Yagi, 1991; Adriano Costa de and Renan da Silva, 2019; Soares et al., 2021).

Chile is characterized by a variety of ecosystems that goes from the desert in the northern part of the country to the temperate Valdivian rainy forest in the south. This work comprises the study of beehives located near the vegetation of the Valdivian temperate forest that dominates as an ecoregion located in the Southern region of Chile characterized by rainy weather and perennial forests that are home to exclusive fauna and flora, like the ancient “araucarias” (*Araucaria araucana* (Molina) K.Koch) and “alerces” (*Fitzroya cupressoides* (Molina) I.M.Johnst.). The main difference between the tropical jungles and this Chilean rainforest, is that the latter grows in cold climates with very strict winters, making it a natural global heritage. The Valdivian temperate forest is characterized by its extraordinary endemism, nearly 90% at the species level and 34% at the genus level for woody species (Veblen and Schlegel, 1982; Marticorena, 2009). Some of the important species as available source of pollen for *Apis mellifera* are “arrayán” (*Luma apiculata* (DC.) Burret), “avellano” (*Gevuina avellana* Molina), “coigüe” or “coihue” (*Nothofagus dombeyi* (Mirb.) Oerst.), “colihue” (*Chusquea culeou* É. Desv.), “copihue” (*Lapageria rosea* Ruiz & Pav.), “luma” (*Amomyrtus luma* (Molina) D. Legrand & Kausel), “murta” (*Ugni molinae* Turcz.), “notro” (*Embothrium coccineum* J.R. Forst. & G. Forst.), “tineo” (*Weinmannia trichosperma* Cav.), “ulmo” (*Eucryphia cordifolia* Cav.) and “matico” (*Buddleja globosa* Hope) (Marticorena, 1990; Marticorena, 2009).

This is the first characterization of several HBP from the south zone of Chile according to the botanical origin, polyphenolic profile, and antioxidant capacity. Selected HBPs were evaluated for bioaccessibility using a model for the human gastrointestinal tract. Total phenolic contents, antioxidant capacity, and recovery index of quercetin, myricetin, and cinnamic acid were determined in the different steps of the *in vitro* gastrointestinal digestion. Likewise, this is the first report addressing the protective effect of *in vitro* digested HBP towards DNA damage induced by peroxyl radicals.

## 2 Materials and Methods

### 2.1 Chemicals and Reagents

The compounds 6-hydroxy-2,5,8-tetramethylchroman-2-carboxylic acid (Trolox), fluorescein disodium salt (FL), Folin-Ciocalteu’s phenol reagent, 2,2′-azo-bis(2-amidinopropane) dihydrochloride (AAPH), 2,4,6-tri(2-pyridyl)-S-triazine (TPTZ), sodium bicarbonate (NaHCO_3_), potassium chloride (KCl), sodium chloride (NaCl), calcium chloride dihydrate (CaCl_2_·2H_2_O), dibasic potassium phosphate (K_2_HPO_4_), mucin, pepsin, α-amylase, pancreatin, bile salts and all standards of compounds studied were purchased from Sigma-Aldrich (St. Louis, MO, United States). Aluminium chloride (AlCl_3_) and ferric chloride (FeCl_3_) were supplied by Merck (Darmstadt, Germany). All solvents were high-performance liquid chromatography (HPLC) grade. Water was purified in a Milli-Q system (Synergy, Millipore, Darmstadt, Germany).

### 2.2 Honeybee Pollen Samples (HBP)

Twelve honeybee pollen (HBP) samples from Southern Chile, X Región de Los Lagos (GPS coordinates 41°16′ 40.099″ S, 72°41′ 7968″ W) were provided as vacuum-packed when fresh and were frozen at −20°C by beekeepers. The HBP were collected during the dry seasons of 2018 and 2019. The botanical origin was determined based on pollen grain morphology according to the microscopy method described in Chilean Regulation NCh3255, 2011 (Montenegro et al., 2008). Five grams of each type of bee pollen corbiculae were separated by color, and each fraction was weighed. After this, one corbicula of each type of HBP was wrinkled with alcohol to disperse the pollen grains. Several drops of red dye (Calberla’s solution) were used to stain the grains to allow observation under a light microscope (Avila et al., 1992; Montenegro et al., 2008). To determine the botanical origin, specific literature (Heusser and Moar, 1973; Marticorena, 1990) and the botanical bee pollen catalogue at the Laboratory of Botany (Department of Plant Sciences, Faculty of Agronomy and Forest Engineering, Pontificia Universidad Católica de Chile, Santiago, Chile) were consulted.

### 2.3 Honeybee Pollen Extracts and Phenolic Characterization

One gram of fresh honeybee pollen (HBP) was consecutively extracted thrice with 10 mL aliquots of analytical grade absolute ethanol (EtOH) by ultrasonic extraction (Elmasonic S 10 HELMA) at room temperature (25°C) for 10 min. The mixture was centrifuged at 3,130*g* for 5 min, filtered using Whatman No. 1 paper, and the supernatant collected. The three collected supernatants were combined and adjusted to a final volume of 50 mL with EtOH aimed to obtain a 0.02 g HBP/mL final concentration. HBPEs were stored at −80°C in the dark until use.

The total phenolic content in the extracts was determined by the Folin-Ciocalteu’s method using gallic acid as a standard. The results were expressed as gallic acid equivalents (GAE) per 100 g of HBP (mg GAE/100 g fresh HBP) (Bridi et al., 2019; Oyarzún et al., 2021). The flavonoid content, measured by AlCl_3_ method, was expressed as milligrams of quercetin equivalents (QE) per 100 g of HBP (mg QE/100 g fresh HBP) (Bridi et al., 2019; Oyarzún et al., 2021).

### 2.4 HPLC-DAD Analysis

The polyphenols and abscisic acid identification and quantification in HBPEs were carried out using a Hitachi Chromaster 5000 series HPLC instrument equipped with an autosampler and a photodiode array detector (Hitachi, Tokyo, Japan). The HPLC system was controlled by the Chromaster system manager V1.2. HBPEs (10 μL) were eluted using a mobile phase mixture of (A) methanol, (B) acetonitrile, and (C) 0.1% aqueous formic acid. The gradient elution employed was: 0–10 min 20% B, 80% C; 10.1–40 min 7.5% A, 25% B, 67.5% C; 40.1–50 min 15% A, 25% B, 60% C; 50.1–65 min 15% A, 45% B, 40% C, and returned to starting conditions during the following 15 min. The column used was a 250 mm × 4.6 mm, i.d., Purospher STAR RP-18 end-capped with a guard column of the same type (Merck, Darmstadt, Germany). The flow rate was 0.8 mL/min and the oven column was set at 35°C. The absorbance of the eluate was monitored in the 210–550 nm range using a diode array detector (DAD) and the chromatograms were integrated for all standards and HBPEs at 290 nm. Phenolic compounds identification was performed by comparison of the retention times exhibited by the standards and UV−vis spectra. For quantification, a multistandard combination was used in equal concentrations of each polyphenol (range 5–250 μM) to obtain calibration curves of all standards studied. Detection limits of standards ranged between 2 and 133 μg/g in HBP. All analyses were performed in triplicate for standards and HBPEs (Bridi et al., 2019; Oyarzún et al., 2021).

### 2.5 Ferric Reducing Antioxidant Potential

The FRAP of the HBPEs was determined as previously described by literature (Bridi et al., 2019; Oyarzún et al., 2021). The absorbance was read at 594 nm using a Cytation 5 multimode microplate reader from BioTek Instruments, Inc. (Winooski, VT, United States). As controls, an ethanol solution and Trolox (5–30 μM) were used. The results were expressed as μmol Trolox equivalents per g of HBP (μmol TE/g). Values were reported as means ± SD of three independent determinations.

### 2.6 Oxygen Radical Absorbance Capacity

The ORAC of HBPEs against peroxyl radicals was measured by using the ORAC-fluorescein (ORAC-FL) method according to the literature (Ou et al., 2001) and adapted to fluorescent microplate reader (Cytation 5 from BioTek Instruments Inc.) (Bridi et al., 2019). The fluorescein consumption was measured by the decline in fluorescence intensity (excitation 493 nm; emission 515 nm). AAPH (10 mM) was used as the peroxyl radical producer at 37°C and μM Trolox was used as a standard (2–10 μM). The results were expressed as μmol Trolox equivalents per 100 g of HPB (μmol TE/100 g) and reported as means ± SD of three independent determinations.

### 2.7 *In Vitro* Gastrointestinal Digestion

#### 2.7.1 Preparation of Aqueous Honeybee Pollen Extract

To 50 mL of ethanolic extract (0.02 g HBP/mL EtOH), 10 mL of ultrapure water aliquot was added and ethanol was removed by rotary evaporation (38 ± 2°C). The final volume was adjusted to 10 mL with pure water to obtain an aqueous honeybee pollen extract (HBPEaq) with a final concentration of 0.1 g HBP/mL H_2_O. HBPEaq were stored at −80°C in the dark until analysis.

#### 2.7.2 *In Vitro* Gastrointestinal Digestion Assay

The *in vitro* gastrointestinal digestion assay, simulating the physiological state in the upper digestive tract (mouth, stomach, and small intestine) was performed according to a procedure described in literature (Ah-Hen et al., 2018) with minor modifications. The digestion process is comprised of salivary pre-digestion (MTH), gastric digestion initial (GDI), gastric digestion final (GDF) and small intestinal digestion steps. The latter digestion step was subclassified by their respective small intestinal portion as follows: duodenal (DDM), jejunal (JJM) and ileal (ILN). For the salivary digestion step, the HBPEaq (10 mL) or purified water (control) was mixed with 3 mL of artificial saliva, composed of double-distilled water, 5.21 mg/mL NaHCO_3_, 0.88 mg/mL NaCl, 0.48 mg/mL KCl, 0.33 mg/mL CaCl_2_, 1.04 mg/mL K_2_HPO_4_, 2.16 mg/mL of mucin and 0.1 mg/mL of α-amylase, and adjusted to a pH of 6.8 with 0.1 M HCl. The mixture of sample or control and artificial saliva was homogenized with 20 mL of pure water for 3 min to simulate mastication. MTH digesta (2 mL) was collected and put on ice for 10 min to stop the enzymatic activity. To simulate gastric digestion, 148 mg of pepsin (250 U) dissolved in 2.5 mL of pure water was added immediately to the simulated bolus of salivary digestion, and pH was adjusted to 2.0 with 6 M HCl. The mixture was then incubated at 37°C using a shaking water bath (Labtech, LSB-015S, Italy) at 250 rpm for 2 h. GDI digesta (2 mL) was withdrawn and put in ice for 10 min to stop the enzymatic activity. After the gastric digestion, the small intestinal digestion was simulated, adjusting pH to 6.5 with 0.5 M NaHCO_3_ before 2.5 mL of a mixture of pancreatin (4.0 mg/mL) and bile salts (50.0 mg/mL) (1:1; v/v), dissolved in 15 mL of water, were added and incubated at 37°C in the shaking water bath at 250 rpm for 2 h. The sample was withdrawn after adjusting pH to 6.5 (DDM), after the first (JJM) and second hour (ILN) of the digestion process. At each step of digestion (at different time intervals), 2 mL aliquots of the obtained extract or control were withdrawn for analysis, cooling the test tubes in ice for 10 min to stop the enzymatic activity. The supernatants (bioaccessible fractions) were used for the analysis of total phenolic content (Folin-Ciocalteu), antioxidant activity (ORAC-FL), and quercetin, myricetin, and cinnamic acid concentration by HPLC-DAD. For the Folin–Ciocalteu and ORAC-FL tests, the values found for the controls were subtracted from the HBPEaqs. Before the HPLC analysis, these aliquots were sonicated at 4°C in an ice bath for 30 min. Subsequently, they were centrifuged for 10 min at 10,845 *g* to facilitate the separation of the components and purification of the sample. Subsequently, the supernatant was collected and filtered on a 0.22 μm cellulose acetate pore filter and then quantified (Gonçalves et al., 2019).

The bioaccessibility index (BI) was calculated as the percentage of the tested compound remaining in the bioaccessible fraction related to the original non-digested sample (Eq. 1).

BI=[CDS/CFS]×100 (1)

CDS is the concentration of the bioactive or its antioxidant activity at the end of a digestion step and CFS is the concentration of the antioxidant activity of the same bioactive in the sample as determined by a chemical extraction procedure.

#### 2.7.3 Inhibition of Peroxyl Radical Induced Supercoiled Plasmid DNA Strand Breakage

The inhibitory effect against DNA damage induced by peroxyl radicals (de Camargo et al., 2014) was tested with phenolics recovered from HBPE12 subjected to *in vitro* digestion. In this, the lyophilized phenolic extracts recovered from each phase of digestion were diluted in phosphate buffer saline solution (PBS) (1:10, m/v) and transferred to Eppendorf tubes (2 µL) and mixed with 2 µL PBS 10 mmol/L, pH 7.4, followed by the addition of supercoiled plasmid DNA pBR 322 from *Escherichia coli* RRI (2 µL) diluted in PBS (50 μL/mL), and 7 mM AAPH solution (4 µL). After incubation at 37°C in the dark for 1 h, loading dye (0.25% bromophenol blue, 0.25% xylene cyanol, 50% glycerol in distilled water) was added (1 µL). The mixture was loaded onto 0.7 (w/v) agarose gel prepared in buffer Tris−acetic acid−EDTA (40 mM Tris-acetate and 1 mM EDTA, pH 8.5). SYBR safe (100 μL/L) was used to stain the gel. The experiment was carried out at 80 V for 90 min employing submarine gel electrophoresis equipment (VWR, Radnor, PA, United States). A Sony digital camera under UV light was used to acquire the images which were analyzed using AlphaEase stand-alone software (Alpha Innotech Co., San Leandro, CA, United States). The percentage of inhibition was calculated according to Eq. 2. Supercoiled DNA retention was expressed as a percentage.

DNA strand breakage inhibition=[(supercoiled DNA intensity in the presence of oxidant and extract/supercoiled DNA intensity devoid of oxidant and extract)]×100 (2)

### 2.8 Statistical Analysis

Data represented as the mean ± SD (standard deviation). Statistical analysis of the data was performed with one-way ANOVA followed by Tukey post-hoc test. All analyses were performed using Origin Pro 8 software (MA, United States). Correlation analysis was carried out using Pearson correlation analysis. Differences were considered significant at *p* < 0.05.

## 3 Results and Discussion

### 3.1 Botanical Origin

The botanical origin of the bee pollen analyzed is presented in Table 1. Botanical origin describes the plant sources used by *Apis mellifera* bees to produce HBP. This report allows to categorize species according to their geographical distribution as native, non-native, or mixed and classified as monofloral or multifloral depending on the botanical composition of the samples. Monofloral bee pollen has not less than 80% of the same species (taxon) and multifloral is a mixture of pollen from different taxa where no taxon constitutes more than 80% (Campos et al., 2008). All analyzed HBPs from the X Region de Los Lagos of Chile corresponded to non-native multifloral and the predominant plant species were *Brassica rapa* L., *Lotus pedunculatus* Cav., and *Ulex europaeus* L. The native species appears as important minor species (3–15%) as “matico” (*Buddleja globosa* Hope), “arrayán” (*Luma apiculata* (DC.) Burret), “notro” (*Embothrium coccineum* J.R. Forst. & G. Forst.), and “ulmo” (*Eucryphia cordifolia* Cav.). Honeybee is selective in the use of the species that provide pollen. The group of introduced species that appear in a significant frequency in the HBPs studied are also more abundant in this geographical area. In addition, they are sources highly appreciated by bees for pollen collection.

**TABLE 1:** Palynological analysis of the botanical origin and classification of honeybee pollen samples (HBPs).

Sample	Classification	Predominant species (≥45%)	Secondary species (16–45%)		Important minor species (3–15%)		Minor species (≤3%)	
HBP1	Multifloral Non-Native		*Brassica rapa* L.	16.78	*Acacia* sp.	14.82		
					*Taraxacum officinale* F.H.Wigg.	14.82		
					*Trifolium repens* L.	11.98		
					Buddleja globosa Hope	11.11		
					*Ulex europaeus* L.	8.49		
					*Embothrium coccineum* J.R.Forst. & G.Forst.	8.06		
					*Gaultheria mucronata* (L.f.) Hook. & Arn.	7.84		
					*Tepualia stipularis* (Hook.fil.) Griseb.	6.10		
HBP2	Multifloral Non-Native		*Trifolium repens* L.	16.30	*Luma apiculata* (DC.) Burret	12.41		
					*Caldcluvia paniculata* (Cav.) D. Don	11.85		
					*Eucryphia cordifolia* Cav.	11.30		
					*Buddleja globosa* Hope	10.19		
					*Brassica rapa* L.	8.89		
					*Taraxacum officinale* F.H. Wigg.	8.89		
					*Tepualia stipularis* (Hook. & Arn.) Griseb.	8.33		
					*Castanea sativa* Mill.	7.22		
					*Gaultheria mucronata* (L.f.) Hook. & Arn.	4.63		
HBP3	Multifloral Non-Native		*Lotus pedunculatus* Cav.	23.05	*Brassica rapa* L.	14.86		
					*Luma apiculata* (DC.) Burret	12.95		
					*Taraxacum officinale* F.H. Wigg.	11.24		
					*Buddleja globosa* Hope	7.62		
					*Embothrium coccineum* J.R.Forst. & G.Forst.	7.43		
					*Rubus constrictus* P.J.Müll. & Lefèvre	7.24		
					*Ulex europaeus* L.	6.86		
					*Trifolium repens* L.	5.33		
HBP4	Multifloral Non-Native		*Brassica rapa* L.	17.44	*Castanea sativa* Mill.	3.43	*Embothrium coccineum*	2.41
			*Ulex europaeus* L.	17.03	*Lotus pedunculatus* Cav.	15.63	*Gaultheria mucronata*	1.60
					*Taraxacum officinale* F.H. Wigg.	13.62		
					*Trifolium repens* L.	10.82		
					*Luma apiculata* (DC.) Burret	8.22		
					*Rubus constrictus* P.J.Müll. & Lefèvre	8.22		
					*Buddleja globosa* Hope	5.01		
HBP5	Multifloral Non-Native		*Caldcluvia paniculata* (Cav.) D. Don	22.93	*Rubus constrictus* P.J.Müll. & Lefèvre	14.65		
					*Eucryphia cordifolia* Cav.	13.59		
					*Brassica rapa* L.	11.04		
					*Lotus pedunculatus* Cav.	9.98		
					*Trifolium repens* L.	8.49		
					*Taraxacum officinale* F.H. Wigg.	8.07		
					*Buddleja globosa* Hope	6.58		
					*Fern spores*	4.67		
HBP6	Multifloral Non-native		*Brassica rapa* L.	17.42	*Eucryphia cordifolia* Cav.	15.98	*Fern spores*	0.21
					*Trifolium repens* L.	13.73		
					*Buddleja globosa* Hope	13.73		
					*Rubus constrictus* P.J.Müll. & Lefèvre	12.09		
					*Lotus pedunculatus* Cav.	9.84		
					*Taraxacum officinale* F.H. Wigg.	9.84		
					*Luma apiculata* (DC.) Burret	7.17		
HBP7	Multifloral Non-native				*Ulex europaeus* L.	14.72		
					*Acacia* sp.	13.96		
					*Castanea sativa* Mill.	13.02		
					*Weinmannia trichosperma* Cav.	11.51		
					*Buddleja globosa* Hope	10.57		
					*Trifolium repens* L.	10.38		
					*Taraxacum officinale* F.H. Wigg.	7.36		
					*Lotus pedunculatus* Cav.	5.85		
					*Brassica rapa* L.	5.47		
					*Luma apiculata* (DC.) Burret	3.96		
					*Rubus constrictus* P.J.Müll. & Lefèvre	3.21		
HBP8	Multifloral Non-native		*Brassica rapa* L.	22.03	*Luma apiculata* (DC.) Burret	13.03		
			*Lotus pedunculatus* Cav.	18.58	*Buddleja globosa* Hope	12.84		
					*Ulex europaeus* L.	12.64		
					*Taraxacum officinale* F.H. Wigg.	8.62		
					*Castanea sativa* Mill.	6.71		
					*Fern spores*	5.56		
HBP9	Multifloral Non-native		*Lotus pedunculatus* Cav.	19.08	*Trifolium repens* L.	12.91		
			*Ulex europaeus* L.	17.15	*Taraxacum officinale* F.H. Wigg.	9.83		
					*Brassica rapa* L.	9.06		
					*Luma apiculata* (DC.) Burret	7.71		
					*Gaultheria mucronata* (L.f.) Hook. & Arn.	7.51		
					*Acacia* sp.	6.94		
					*Castanea sativa* Mill.	5.20		
					*Buddleja globosa* Hope	4.62		
HBP10	Multifloral Non-native		*Brassica rapa* L.	20.21	*Caldcluvia paniculata* (Cav.) D. Don	14.53	*Gaultheria mucronata*	2.53
					*Embothrium coccineum* J.R. Forst. & G. Forst.	13.05		
					*Tepualia stipularis* (Hook. & Arn.) Griseb.	12.63		
					*Luma apiculata* (DC.) Burret	11.58		
					*Eucryphia cordifolia* Cav.	10.11		
					*Lotus pedunculatus* Cav.	7.79		
					*Castanea sativa* Mill.	7.58		
HBP11	Multifloral Non-native		*Brassica rapa* L.	27.23	*Tepualia stipularis* (Hook. & Arn.) Griseb.	15.11	*Gaultheria mucronata*	1.7
			*Lotus pedunculatus* Cav.	17.23	*Buddleja globosa* Hope	9.57		
					*Castanea sativa* Mill.	9.57		
					*Trifolium repens* L.	8.72		
					*Embothrium coccineum* J.R. Forst. & G. Forst.	5.75		
					*Eucryphia cordifolia* Cav.	5.11		
HBP12	Multifloral Non-native		*Brassica rapa* L.	26.98	*Tepualia stipularis* (Hook. & Arn.) Griseb.	14.36		
					*Buddleja globosa* Hope	13.66		
					*Trifolium repens* L.	11.58		
					*Rubus constrictus* P.J.Müll. & Lefèvre	10.68		
					*Acer campestre* L.	9.64		
					*Ulex europaeus* L.	9.09		
					*Castanea sativa* Mill.	4.02		

### 3.2 Total Phenolic and Flavonoid Contents and Antioxidant Capacity

Table 2 shows the mean values of total phenolic content (TPC), total flavonoids contents (TFC), FRAP, and ORAC-FL, in HBPE. HBPE3 showed the highest value for TPC (1,532 ± 91 GAE/100 g of HBP) while HBPE8 showed the lowest value for TPC (519 ± 16 mg GAE/100 g of HBP). The TFC ranged between 788 ± 4 QE/100 g of HBP (HBPE10) and 215 ± 8 mg QE/100 g HBP (HBPE12). The values of antioxidant capacity, evaluated by FRAP and ORAC-FL, were between 113.99 ± 4.92 (HBPE3) and 24.66 ± 1.23 μmol TE/g HBP (HBPE7), and between 484.98 ± 35.55 (HBPE10) and 176.07 ± 24.20 μmol TE/g HBP (HBPE8), respectively.

**TABLE 2:** Average values of total phenolic content (TPC), total flavonoids content (TFC), FRAP, and ORAC-FL in honeybee pollen extracts (HBPEs).

Sample	TPC (mg GAE/100 g HBP)	TFC (mg QE/100 g HBP)	FRAP (μmol TE/g HBP)	ORAC (μmol TE/g HBP)
HBPE1	1,408 ± 96	493 ± 35	111.25 ± 3.77	377.40 ± 28.32
HBPE2	1,198 ± 54	424 ± 21	103.03 ± 3.04	322.24 ± 20.87
HBPE3	1,532 ± 91	421 ± 46	113.99 ± 4.92	376.34 ± 31.19
HBPE4	1,327 ± 88	444 ± 39	108.54 ± 6.55	385.50 ± 35.34
HBPE5	546 ± 28	422 ± 17	27.52 ± 1.81	177.73 ± 8.81
HBPE6	644 ± 7	465 ± 32	29.71 ± 1.26	205.81 ± 16.92
HBPE7	533 ± 9	408 ± 1	24.66 ± 1.23	179.57 ± 21.80
HBPE8	519 ± 16	397 ± 11	24.96 ± 0.86	176.07 ± 24.20
HBPE9	1,313 ± 40	778 ± 37	26.67 ± 2.33	451.18 ± 31.57
HBPE10	1,438 ± 47	788 ± 4	28.11 ± 2.73	484.98 ± 35.55
HBPE11	1,447 ± 51	748 ± 42	28.83 ± 5.72	480.37 ± 38.57
HBPE12	1,140 ± 55	215 ± 8	93.62 ± 7.71	338.30 ± 24.23

HBP, honeybee pollen, Values are reported as mean ± SD of 3 independent experiments performed in triplicate. The total phenol results are expressed as mg gallic acid equivalents (GAE)/100 g fresh HBP; flavonoids are expressed as mg quercetin equivalents (QE)/100 g fresh HBP; TRAP as μmol Trolox equivalents (TE)/g fresh HBP, and ORAC-FL as μmol Trolox equivalents/g fresh HBP.

The Pearson correlation analysis of the 12 HBPEs showed a statistically significant strong positive correlation with the antioxidant capacity measured by ORAC-FL and total phenolic content (R = 0.93; *p* ≤ 0.01) and a moderate correlation between the flavonoid content (R = 0.66; *p* ≤ 0.01). Nonetheless, no significant correlation was observed between the phenolic or flavonoid content and FRAP. HBP varies in the content of antioxidants phytochemicals depending upon botanical origin, atmospheric conditions, soil nature, and behavior of the bees (Khalifa et al., 2021). The changes in phytochemical content can be monitored using standardized antioxidant capacity assays like ORAC (Prior, 2015). The ORAC value is still a relevant method in food products, and it is increasingly applied in the area of nutraceuticals. A recent study (de Camargo et al., 2019) demonstrated that ORAC and FRAP values were good predictors of the reduction of the activation of NF-κB in a cell model, which is induced by oxidative stress. The average values of total phenols (TPC: 5–14 mg GAE/100 g HBP), flavonoids (TFC: 2–8 mg QE/100 g HBP), ORAC-FL (177–480 μmol TE/g HBP) and FRAP (25–108 μmol TE/g HBP), in HBPEs from the southern region of Chile are similar to HBPEs from the central region of Chile, as V Region de Valparaiso (TPC: 5–14 mg GAE/100 g HBP; TFC: 1-3 QE/100 g HBP; ORAC: 160–477 μmol TE/g HBP; FRAP: 42–120 μmol TE/g HBP) (Bridi et al., 2019; Oyarzún et al., 2021) and from others countries like Italy (TPC: 13–25 mg GAE/100 g HBP; TFC: 5-15 QE/100 g HBP; ORAC 500–677 μmol TE/g HBP) (Gabriele et al., 2015), Brazil (TPC: 9–21 mg GAE/100 g HBP; TFC: 0.3-19 QE/100 g HBP; ORAC 300–480 μmol TE/g HBP) (De-Melo et al., 2016) and Portugal (TPC: 16–45 mg GAE/100 g HBP; TFC: 4-10 QE/100 g HBP; ORAC: 150–255 μmol TE/g HBP) (Dias et al., 2016). The regions of origin of these pollens are located in a climatic type known as temperate or one of the subtypes of temperate. Beekeeping products from temperate climates are characterized by the abundance of phenolic compounds.

### 3.3 Polyphenolic Profiles of HBPEs

The polyphenolic profiles of HBPEs were analyzed by HPLC-DAD. The concentrations of the most characteristic phenolic compounds (cinnamic acids, flavonols, flavones, and flavanones) were determined. The results are shown in Tables 3, 4 and Supplementary Figures S1, S2. Sixteen phenolic compounds and abscisic acid were quantified, among them five phenolic compounds were well-established in all HBPEs (ferulic acid, cinnamic acid, rutin, myricetin, and quercetin). The concentrations of ferulic and cinnamic acids ranged from 5.82 to 43.6 and from 3.30 to 29.5 mg/100 g HBP, respectively. Sinapic acid, syringic acid, chlorogenic acid, apigenin, naringin, and abscisic acid were also found in most HBPEs in significant quantities. In contrast, rhamnetin was detected only in two HBPEs. The abscisic acid was found in a concentration range from 2.6 to 26.8 mg/100 g of HBP. The presence of abscisic acid, an important phytohormone regulating plant growth, is implicated in the responses of the plants to a variety of stresses (Sharma and Nayyar, 2016). The range of values found may be related to the period (2018–2019) or location where the HBPEs were collected.

**TABLE 3:** Phenolic acids and abscisic acid of honeybee pollen extracts (HBPEs) as determined by HPLC-DAD.

mg/100 g HBP								
Sample	Chlorogenic acid	Caffeic acid	Syringic acid	Coumaric acid	Sinapic acid	Ferulic acid	Abscisic acid	Cinnamic acid
HBPE1	3.03 ± 0.36	4.48 ± 1.76	0	4.09 ± 0.24	62.13 ± 0.79	10.76 ± 0.36	35.52 ± 0.6	16.36 ± 0.29
HBPE2	3.61 ± 1.09	0	8.78 ± 0.10	3.06 ± 0.15	0	30.19 ± 0.42	16.62 ± 0.55	24.11 ± 1.06
HBPE3	3.93 ± 0.07	0	9.02 ± 0.04	4.31 ± 0.01	24.78 ± 2.57	43.6 ± 1.00	26.81 ± 0.76	29.30 ± 1.05
HBPE4	5.07 ± 0.36	0	9.10 ± 0.10	3.60 ± 0.11	1.28 ± 0.07	18.22 ± 1.63	20.99 ± 0.18	25.47 ± 0.28
HBPE5	1.29 ± 0.12	0	10.02 ± 0.20	0	14.07 ± 0.48	8.31 ± 0.77	0	3.30 ± 0.08
HBPE6	2.10 ± 0.44	0	14.29 ± 0.14	0	14.45 ± 0.04	11.05 ± 0.24	3.51 ± 0.06	4.16 ± 0.03
HBPE7	0	0	10.14 ± 0.05	0	10.42 ± 0.11	6.78 ± 1.24	2.56 ± 0.12	4.48 ± 0.08
HBPE8	0	0	12.06 ± 0.20	0.03 ± 0.04	11.98 ± 0.14	9.97 ± 0.73	3.21 ± 0.11	4.62 ± 0.07
HBPE9	2.42 ± 0.07	1.72 ± 0.01	0	1.61 ± 0.02	0	14.13 ± 0.19	11.56 ± 0.18	12.38 ± 0.04
HBPE10	0.38 ± 0.13	4.2 ± 0.01	0	2.29 ± 0.05	0	20.39 ± 0.22	11.72 ± 0.25	14.78 ± 0.14
HBPE11	0.64 ± 0.04	0	4.68 ± 0.08	0.21 ± 0.04	0	9.57 ± 0.17	18.88 ± 0.32	19.25 ± 0.38
HBPE12	0	0	18.85 ± 0.36	0.81 ± 0.03	0	5.82 ± 0.31	18.97 ± 0.16	29.5 ± 0.16

HBP, honeybee pollen, Data are expressed as mg/100 g fresh HBP, and the values represent the means ± SD (*n* = 3).

**TABLE 4:** Flavonoids of honeybee pollen extracts (HBPEs) as determined by HPLC-DAD.

mg/100 g HBP									
Sample	Epicatechin	Rutin	Myricetin	Quercetin	Naringenin	Apigenin	Kaempferol	Rhamnetin	Galangin
HBPE1	72.01 ± 3.47	12.26 ± 1.61	155.53 ± 14.3	44.72 ± 0.44	0	98.69 ± 2.85	0	0.58 ± 0.31	0
HBPE2	28.43 ± 0.04	6.14 ± 0.93	476.39 ± 4.73	50.46 ± 6.94	12.39 ± 0.14	100.22 ± 13.92	0	0	0
HBPE3	41.41 ± 4.33	6.92 ± 1.90	689.24 ± 12.76	51.92 ± 1.69	16.17 ± 0.58	125.14 ± 1.55	0	0	0
HBPE4	35.97 ± 3.53	6.34 ± 0.07	68.14 ± 0.93	65.56 ± 1.98	11.57 ± 0.04	88.60 ± 6.29	0	0	0
HBPE5	0	90.54 ± 0.69	24.07 ± 1.13	6.53 ± 3.42	2.19 ± 0.53	10.29 ± 1.86	3.73 ± 0.61	0	2.86 ± 0.77
HBPE6	0	74.79 ± 0.55	27.06 ± 0.68	18.62 ± 0.88	3.37 ± 0.18	7.46 ± 0.53	0.5 ± 0.06	0	1.11 ± 0.13
HBPE7	199.06 ± 1.71	57.62 ± 0.19	26.06 ± 0.68	5.40 ± 0.58	2.30 ± 0.03	5.84 ± 1.80	1.84 ± 0.46	0	2.06 ± 0.30
HBPE8	0	78.27 ± 0.40	24.33 ± 1.13	12.97 ± 1.33	2.39 ± 0.32	0	1.99 ± 0.09	0	4.04 ± 0.18
HBPE9	0	1.78 ± 0.04	10.07 ± 0.20	19.50 ± 0.31	0	3.29 ± 0.15	0	0	0
HBPE10	0	2.72 ± 1.33	17.00 ± 0.29	30.52 ± 0.67	0.29 ± 0.01	0	0	0	0
HBPE11	5.24 ± 0.44	3.69 ± 0.09	16.47 ± 0.12	24.20 ± 1.06	3.37 ± 0.37	13.69 ± 1.40	0.42 ± 0.32	0	1.93 ± 0.05
HBPE12	4.42 ± 0.13	2.58 ± 0.16	48.79 ± 2.02	109.85 ± 4.78	0.71 ± 0.06	7.14 ± 0.12	20.41 ± 0.18	0	0

HBP, honeybee pollen, Data are expressed as mg/100 g fresh HBP, and the values represent the means ± SD (*n* = 3).

The content of rutin, which is a quercetin derivative, varied from 1.14 to 90.54 mg/100 g HBP while the concentration of aglycone form ranged from 5.40 to 109.85 mg/100 g HBP. It is well accepted that flavonoid aglycones generally exhibit higher antioxidant activity than that of their conjugated form. Furthermore, although an extremely high concentration of myricetin was found (10.07–689.24 mg/100 g HBP), this compound easily undergoes autoxidation and is less stable than quercetin, which could lead to its underestimation during long-term storage, thus not necessarily reflecting the initial concentration in the feedstock (Atala et al., 2017). Quercetin has been found to exhibit higher antioxidant activity and can be used in food and biological systems to promote health and reduce disease risks (Huber et al., 2009; Oh et al., 2021; Yeung et al., 2021). Therefore, literature data and the results presented here lend support to the proposal to use quercetin as a marker for determining the quality of Chilean HBP (Bridi et al., 2019). Furthermore, it presented a higher antioxidant activity towards copper-induced low-density lipoprotein oxidation than several quercetin derivatives (Oh et al., 2021).

### 3.4 *In Vitro* Gastrointestinal Digestion Assay

From the results shown in Table 4, for the phenolic profile of HBP from the X Region of Chile, three HBPEs were selected according to their content of quercetin, to create three categories: high (HBPE12), medium (HBPE10), and low (HBPE5) quercetin content. These HBPEs were used in the static *in vitro* gastrointestinal digestion assay. The impact of gastrointestinal digestion on total phenolic content (TPC), antioxidant activity (ORAC-FL), and quercetin, myricetin, and cinnamic acid concentrations of HBPEaq are shown in Table 5.

**TABLE 5:** Total phenolic content (TPC), antioxidant activity (ORAC-FL), quercetin, myricetin, and cinnamic acid concentration at different digestion steps in the bioaccessible fractions in honeybee pollen extracts (HBPEs).

	HBPE5 (low QE)		HBPE10 (medium QE)		HBPE12 (high QE)	
	TPC (mg GAE/L extract)	Bioaccessibility (%)	TPC (mg GAE/L extract)	Bioaccessibility (%)	TPC (mg GAE/L extract)	Bioaccessibility (%)
HBPEaq*	473 ± 85^a^	100.0	1,561 ± 127^a^	100.0	1,209 ± 34^a^	100.0
MTH	430 ± 37^a^	90.9	1,199 ± 132^b^	76.8	1,021 ± 55^b^	84.4
GD	472 ± 47^a^	100.0	1,176 ± 47^b^	75.3	790 ± 110^c^	65.3
DDM	566 ± 68^a,b^	119.7	1,333 ± 154^b^	85.4	967 ± 129^b^	80.0
JJM	597 ± 69^b^	126.2	1,405 ± 242^ab^	90.0	1,243 ± 58^a^	102.8
ILN	483 ± 78^a^	102.1	1,444 ± 206^ab^	92.5	1,219 ± 71^a^	100.8
	ORAC-FL (μmol TE/g extract)	Bioaccessibility (%)	ORAC-FL (μmol TE/g extract)	Bioaccessibility (%)	ORAC-FL (μmol TE/g extract)	Bioaccessibility (%)
HBPEaq*	167 ± 3^a^	100.0	420 ± 15^a^	100.0	331 ± 22^a^	100.0
MTH	148 ± 9^b^	88.6	370 ± 31^b^	88.1	324 ± 97^b^	82.6
GD	106 ± 9^c^	63.5	303 ± 36^c^	72.1	192 ± 3^c^	37.2
DDM	143 ± 12^b^	85.6	374 ± 39^b^	89.0	314 ± 4^b^	51.9
JJM	149 ± 10^b^	89.2	375 ± 3^b^	89.3	321 ± 81^a^	139.7
ILN	154 ± 13^a,b^	92.2	394 ± 28^ab^	93.8	306 ± 80	125.2
	Quercetin Concentration (mg/100 g HBP)	Bioaccessibility (%)	Quercetin Concentration (mg/100 g HBP)	Bioaccessibility (%)	Quercetin Concentration (mg/100 g HBP)	Bioaccessibility (%)
HBPEaq*	3.67 ± 0.18^a^	100.0	17.84 ± 0.54^a^	100.0	54.92 ± 1.49^a^	100.0
MTH	0.41 ± 0.08^b^	11.1	3.58 ± 0.03^b^	20.1	5.64 ± 0.08^b^	10.3
GD	0.31 ± 0.00^b^	8.4	2.92 ± 0.29^b^	16.4	7.61 ± 0.19^b^	13.8
DDM	2.21 ± 0.28^c^	60.2	4.12 ± 0.09^bc^	23.1	7.29 ± 3.78^b^	13.3
JJM	2.42 ± 0.32^c^	65.9	4.94 ± 0.60^c^	27.7	8.58 ± 2.29^b^	15.6
ILN	2.25 ± 0.36^c^	61.3	4.95 ± 0.75^c^	27.7	8.52 ± 2.21^b^	15.5
	Myricetin Concentration (mg/100 g HBP)	Bioaccessibility (%)	Myricetin Concentration (mg/100 g HBP)	Bioaccessibility (%)	Myricetin Concentration (mg/100 g HBP)	Bioaccessibility (%)
HBPEaq*	14.49 ± 0.14^a^	100.0	16.25 ± 0.40^a^	100.0	40.58 ± 0.26^a^	100.0
MTH	11.60 ± 1.56^b^	80.1	1.29 ± 0.17^b^	13.5	24.61 ± 2.39^b^	60.6
GD	5.26 ± 1.36^c^	36.3	5.21 ± 0.34^c^	54.5	28.81 ± 4.72^b^	70.9
DDM	21.87 ± 0.43^d^	150.9	10.59 ± 0.06^d^	110.9	36.19 ± 1.51^a^	89.2
JJM	21.80 ± 0.88^d^	150.4	13.81 ± 0.16^e^	144.7	34.91 ± 3.59^ab^	86.0
ILN	21.43 ± 0.67^d^	147.8	13.79 ± 0.29^e^	144.5	36.38 ± 6.80^a^	89.7
	Cinnamic acid Concentration (mg/100 g HBP)	Bioaccessibility (%)	Cinnamic acid Concentration (mg/100 g HBP)	Bioaccessibility (%)	Cinnamic acid Concentration (mg/100 g HBP)	Bioaccessibility (%)
HBPEaq*	1.61 ± 0.10	100.0	13.57 ± 0.12^a^	100.0	36.30 ± 0.31^a^	100.0
MTH	ND	0.0	1.44 ± 0.13^b^	10.6	0.19 ± 0.04^b^	0.5
GD	ND	0.0	1.32 ± 0.19^b^	9.7	0.05 ± 0.00^b^	0.0
DDM	ND	0.0	1.24 ± 0.04^b^	9.1	1.28 ± 0.02^b^	3.47
JJM	ND	0.0	1.51 ± 0.03^b^	11.2	2.04 ± 0.03^b^	5.54
ILN	ND	0.0	1.63 ± 0.05^b^	12.0	2.04 ± 0.03^b^	5.52

HBP, honeybee pollen; HBPEaq* aqueous honeybee pollen extract (non-digested), MTH, oral phase; GD, gastric digestion; DDM, duodenum; JJM, jejunum; ILN, ileum. Identical letters indicate absence of significant difference (ANOVA, Tukey *p* < 0.05).

The TPC of HBPEaq slightly decreased during salivary pre-digestion (MTH) (range recovery 84.4–94.1%) and gastric digestion initial (GDI) (range recovery 65.3–100%) steps in all HBPEaqs. On the other hand, at the end of the intestinal digestion step (JJM) and (ILN) full bioaccessibility of the phenolics was found, showing no significant difference with the HBPEaq (*p* < 0.05). The decrease, despite being low, in the oral phase can be related to the low solubility of phenolic compounds in salivary fluid and the short period of this step (3 min) (Ydjedd et al., 2017). In the stomach, the reactivity of polyphenols with the Folin-Ciocalteu reagent could be affected by acidity pH (pH < 2), with similar results reported in other studies (Wojtunik-Kulesza et al., 2020).

The antioxidant activity of plant food extracts is mainly linked to their phenolic compounds. However, the antioxidant properties of these compounds could change by chemical alterations resulting from different mechanisms during gastrointestinal digestion. The influence of *in vitro* gastrointestinal digestion was evaluated using ORAC-FL method. Regarding the results of HBPEaq after digestion, the average bioaccessibility in the oral phase is 86%, decreasing to 57% in the gastric phase. In intestinal digestion steps more bioaccessibility was found, mainly the last steps (JJM and ILN) with an average of 106 and 103%, respectively, and no significant difference with the HBPEaq (*p* < 0.05). The results obtained in the bioaccessibility of the TPC (Folin-Ciocalteau) and ORAC-FL are comparable and consistent with the correlation described above.

The HPLC-DAD analysis allowed identification and quantification after each digestion phase of quercetin and myricetin as well as cinnamic acid. The HBPEaq analyzed showed different content of quercetin. The results obtained showed variable bioaccessible quercetin after the digestion process. Quercetin bioaccessibility during salivary pre-digestion (MTH) varied within a range from 10 to 20% and during gastric digestion initial (GDI) from 8 to 16%. The detected concentrations of cinnamic acid were very low (range 3–12%) and were not detected, even in HBPE5 which was expected since the original aqueous extract (non-digested) had a very low concentration of cinnamic acid. In contrast, myricetin showed a high bioaccessibility after the digestion process, mainly in the intestinal digestion steps, reaching values greater than 100%. This increase in the number of flavonoids may be related to the hydrolysis of some complex compounds from their glycoside to aglycone form (Chait et al., 2020). The release of individual phenolics during digestion differs from one compound to another. In fact, some phenolic acids (gallic and *p*-coumaric acids) can be released during the gastric phase in an acidic medium. Likewise, some flavonoids (naringenin, quercetin-rhamnoside, and myricetin-rhamnoside) have been reported to be hydrolyzed. Furthermore, other phenolic compounds are released in a neutral medium after oral and intestinal phases (Ydjedd et al., 2017).

The gastrointestinal environment is an important site of prooxidants including caffeine, sulfite myoglobin, dietary nitrite, heme proteins, iron, copper, aldehydes, nonsteroidal anti-inflammatory drugs, polycyclic aromatic hydrocarbons, and mycotoxins (Halliwell et al., 2000; Fuentes et al., 2021; Sampaio et al., 2021; Soares et al., 2021). Therefore, the prevention of oxidative stress at the gastrointestinal level is extremely relevant. The present study anticipates that, regardless of the sample, quercetin and myricetin would reach gastric and intestinal phases. In contrast, cinnamic acid was not detected at any stage of digestion since its initial concentration was the lowest (HBPE5) among all tested materials.

### 3.5 Inhibition of Peroxyl Radical Induced Supercoiled Plasmid DNA Strand Breakage

DNA damage is well recognized by its potential to cause mutagenesis that may lead to cancer initiation, and may be caused by many xenobiotics that induce the generation of ROS. According to a recent report of Ministerio de Salud de Chile (MINSAL, 2018 https://www.gob.cl/plannacionaldecancer/) the mortality from intestinal cancer (small, colon, and rectum) has increased by 49% in Chile, which is higher than that of the prostate (34%) and breast (29%) cancers. Therefore, there is an interest in obtaining natural products that may prevent DNA damage at the gastrointestinal level. In the present study, in general, both TPC and ORAC values were higher in the intestinal phase than the values found after the gastrointestinal digestion. According to literature, TPC (de Camargo et al., 2014; Ayoub et al., 2016) and quercetin concentration (de Camargo et al., 2014) were highly correlated with the inhibition of peroxyl radical-induced DNA oxidation. Furthermore, the protective effect of phenolics obtained from the gastric digesta (78.3%) was lower than those collected from the intestinal digesta (up to 91.2%), which follows the same trend of the antioxidant activity towards peroxyl radicals (ORAC assay, Table 5). Although some differences were found, it is possible to state that the contents of bioaccessible phenolics of HBPEaq are quite effective in preventing DNA damage. The details observed for the electrophoresis and figure of supercoiled plasmid DNA strand breakage inhibition at different digestion steps in the bioaccessible fractions in HBPE12 are presented in Supplementary Figures S3, S4. Therefore, the results of the present study encourage further *in vivo* investigation focusing on the potential of HBPEaq in preventing gastric and/or intestinal cancer.

## 4 Conclusion

The present study is the first to report on HBP from the Southern region of Chile (X Region). The results showed a significant phenolic content and antioxidant capacity and reducing power. Cinnamic acid, myricetin, and quercetin had a high concentration in all HBPEs. Myricetin was the most bioaccessible compound as demonstrated by *in vitro* gastrointestinal digestion, followed by quercetin and cinnamic acid. However, the antioxidant capacity towards ROS in HBPEs remained high in all digestion stages. The same trend was found when evaluating the protective effect of *in vitro* digested HBP towards DNA damage induced by peroxyl radicals, which showed very promising results (up to 91.2% protection). Consequently, HBPs from the X Region de Los Lagos are rich sources of phenolic antioxidants that protect *in vitro* DNA from strand breakage. The latter encourages further investigation focusing on the potential of preventing gastric and/or intestinal cancer.

## Data Availability

The original contributions presented in the study are included in the article/Supplementary Material, further inquiries can be directed to the corresponding authors.
