# Supplementary material for: Honeybee Pollen From Southern Chile: Phenolic Profile, Antioxidant Capacity, Bioaccessibility, and Inhibition of DNA Damage
Source: Front Pharmacol. 2022 Mar 7;13:775219. doi: 10.3389/fphar.2022.775219 (PMC8937017; doi:10.3389/fphar.2022.775219)
Supplement: Supplementary file 1 [file Presentation1.pdf]

Supplementary Material

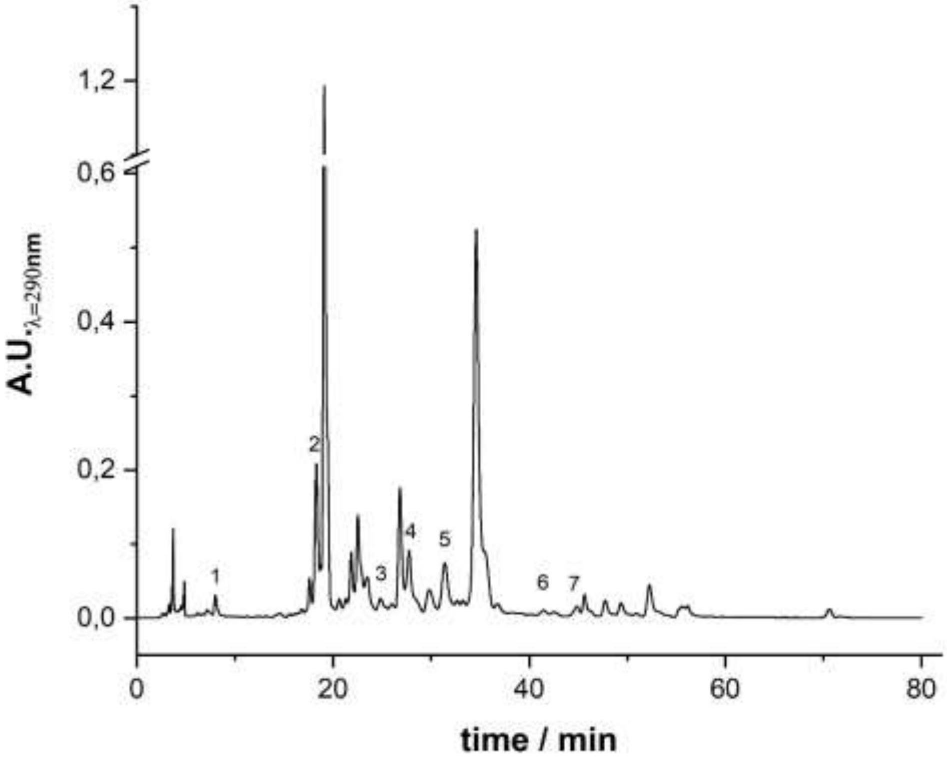

Figure S1. HPLC-DAD chromatogram of phenolic compounds identified in honeybee pollen extract (HBPE12): 1. syringic acid (tR= 8.25); 2. myricetin (tR= 18.55); 3. abscisic acid (tR= 24.75); 4. quercetin (tR= 27.40); 5. cinnamic acid (tR= 31.90); 6. apigenin (tR= 41.40); 7. kaempferol (tR= 44.60).

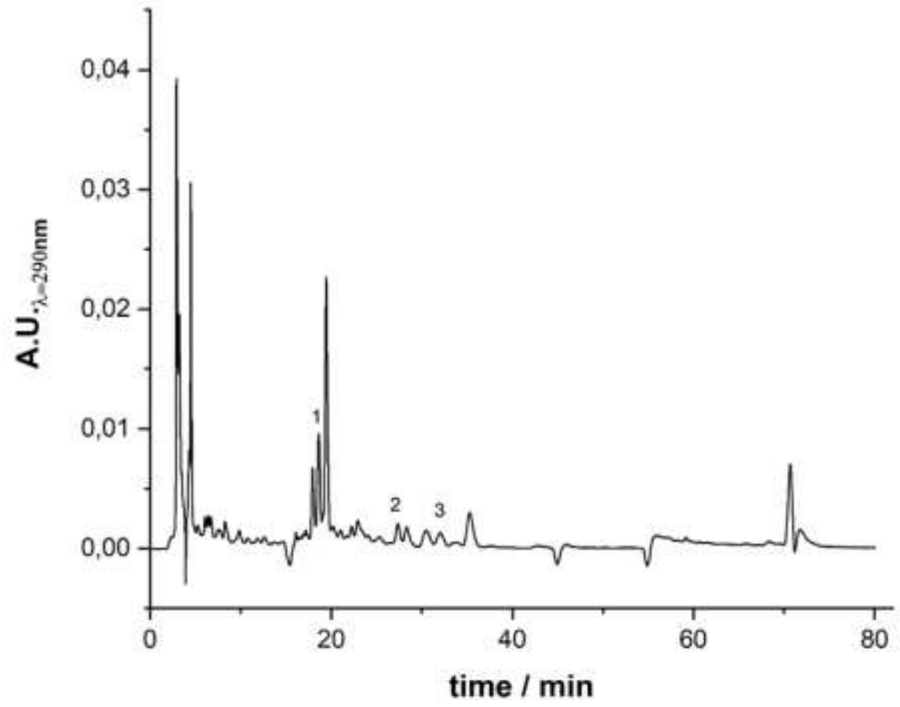

Figure S2. HPLC-DAD chromatogram of phenolic compounds identified in ileum (ILN) digestion step in the bioaccessible fractions in honeybee pollen extract (HBPE12): 1. myricetin (tR= 18.55); 2. quercetin (tR= 27.40); 3. cinnamic acid (tR= 31.90).

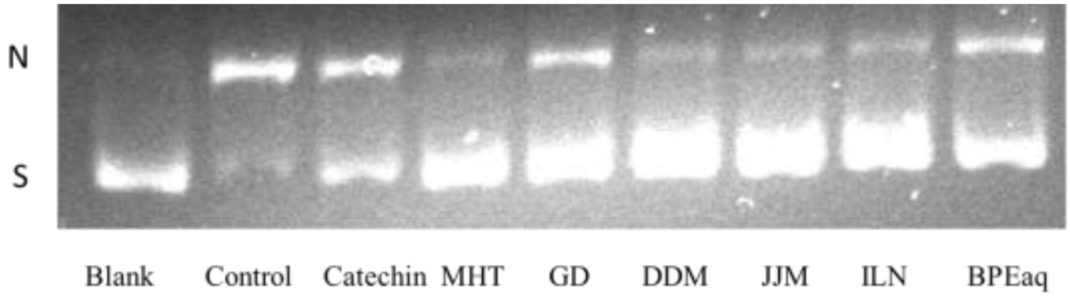

Figure S3. Representative raw data of supercoiled plasmid DNA strand breakage inhibition at different digestion steps in the bioaccessible fractions in honeybee pollen extract (HBPE12). Data represent the mean ± standard deviation of each sample (n = 3). S and N are supercoiled and nicked plasmid DNA strands, respectively. MTH oral phase, GD gastric digestion, DDM duodenum, JJM jejunum, ILN ileum, BPEaq aqueous honeybee pollen extract.

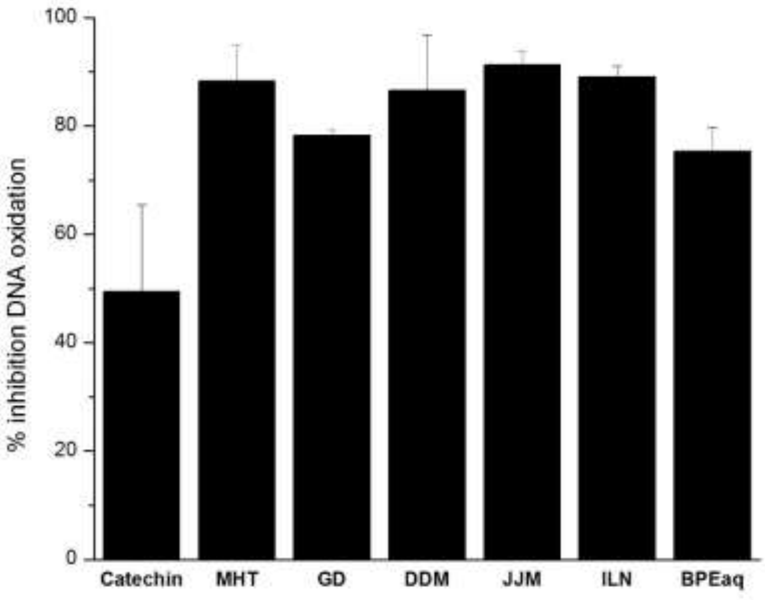

Figure S4. Representative raw data of supercoiled plasmid DNA strand breakage inhibition at different digestion steps in the bioaccessible fractions in honeybee pollen extract (HBPE12). MTH oral phase, GD gastric digestion, DDM duodenum, JJM jejunum, ILN ileum, BPEaq aqueous honeybee pollen extract.
